# The Monoamine Brainstem Reticular Formation as a Paradigm for Re-Defining Various Phenotypes of Parkinson’s Disease Owing Genetic and Anatomical Specificity

**DOI:** 10.3389/fncel.2017.00102

**Published:** 2017-04-18

**Authors:** Stefano Gambardella, Rosangela Ferese, Francesca Biagioni, Carla L. Busceti, Rosa Campopiano, Anna M. P. Griguoli, Fiona Limanaqi, Giuseppe Novelli, Marianna Storto, Francesco Fornai

**Affiliations:** ^1^IRCCS NeuromedPozzilli, Italy; ^2^Department of Translational Research and New Technologies in Medicine and Surgery, University of PisaPisa, Italy; ^3^Department of Biomedicine and Prevention, School of Medicine, University of Rome Tor VergataRome, Italy

**Keywords:** Parkinson’s disease, non-motor symptoms, pyramidal syndrome, genetic Parkinsonism, genotype-phenotype correlation

## Abstract

The functional anatomy of the reticular formation (RF) encompasses a constellation of brain regions which are reciprocally connected to sub-serve a variety of functions. Recent evidence indicates that neuronal degeneration within one of these regions spreads synaptically along brainstem circuitries. This is exemplified by the recruitment of various brainstem reticular nuclei in specific Parkinson’s disease (PD) phenotypes, and by retrospective analysis of lethargic post-encephalitic parkinsonism. In fact, the spreading to various monoamine reticular nuclei can be associated with occurrence of specific motor and non-motor symptoms (NMS). This led to re-consider PD as a brainstem monoamine disorder (BMD). This definition surpasses the anatomy of meso-striatal motor control to include a variety of non-motor domains. This concept clearly emerges from the quite specific clinical-anatomical correlation which can be drawn in specific paradigms of PD genotypes. Therefore, this review article focuses on the genetics and neuroanatomy of three PD genotypes/phenotypes which can be selected as prototype paradigms for a differential recruitment of the RF leading to differential occurrence of NMS: (i) Parkin-PD, where NMS are rarely reported; (ii) LRRK2-PD and slight SNC point mutations, where the prevalence of NMS resembles idiopathic PD; (iii) Severe *SNCA* point mutations and multiplications, where NMS are highly represented.

## Introduction

**Figure 1:**
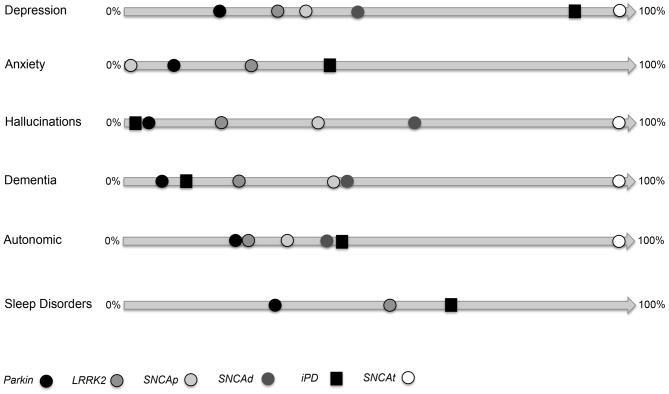
**Non-motor symptoms (NMS) and paradigms of genetic Parkinson’s disease (PD).** This image shows the frequencies of each NMS (depression, anxiety, hallucination, dementia, autonomic dysfunction, sleep disorders) for Parkinsonism related to *Parkin*, *LRRK2*, *SNCA* point mutation (SNCAp), *SNCA* duplication (SNCAd), *SNCA* triplication or homozygote duplication (SNCAt). Image adapted from tables reported in Chaudhuri et al. (2015).

**Figure 2:**
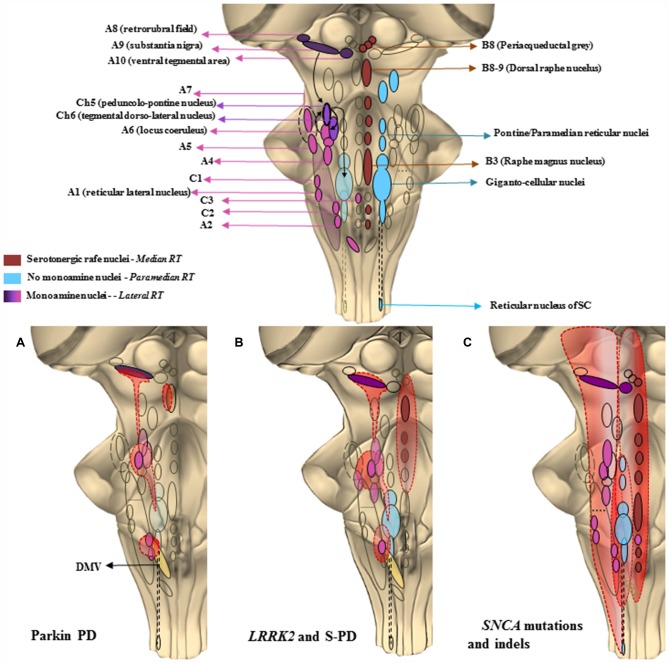
**The chemical neuroanatomy of the brainstem reticular formation (BRF) and its involvement in specific PD paradigms.** This cartoon offers a schematic description of those brainstem areas properly belonging to the reticular formation (RF), which may be the key in PD pathology, as well as their selective recruitment in the three PD paradigms taken into account. In the upper part is shown the constellation of the RF nuclei following a neurotransmitter chemical classification. The isodendritic morphology of the neurons composing the RF nuclei, configures them as crucial stations of both afferent and efferent projections descending and projecting up to the cortex and spinal cord (SC). This network of overlapping connections is involved in a plenty of either extrapyramidal motor and non-motor functions. The major monoamine containing areas, mainly localized in the lateral RF except from C3, are the noradrenergic (A1–A7) adrenergic (C1–C3) dopaminergic (A8–A10) and cholinergic (Ch5–Ch6) nuclei. These are crucial for respiratory activity and for regulating blood pressure and heart rate, micturition, sweat, sleep-wakink cycle as well as descending motor control. Serotonergic nuclei are found in the median RF raphe nuclei, mainly in the B3, B8 and B9 areas. They control vegetative functions such as mood, sleep and sexual behavior, depression and pain. The medial RF, found between the median and the lateral column, is a region lacking monoamine nuclei, but whose giganto-cellular and paramedianpontine nuclei act as a station for fibers connecting with monoamine regions such as A6 (LC) and Ch6. They are involved in voluntary movement regulation, as well as in optical, acoustic and olfactory control due to their connections respectively, to the spinal cord and to the main cranial nerves’ nuclei. In the second part of the figure, it is shown how a progressive and selective anatomic recruitment of such nuclei may be phenotypically and genotypically related to specific PD subtypes. **(A)** Parkin PD—Impairment of ventral SNpc (A9), mild impairment of LC (A6) leading to solely motor symptoms. A minimal alteration of the dorsal raphe nucleus (B8–9) may lead to apathia or anhedonia. DMV is affected as well, which may lead to a partial impairment of the overlapping C2/A2 area controlling the parasympathetic outflow. **(B)** *LRRK2* and slighter *SNCA* point mutations—Featuring the typical PD motor symptoms, along with the presence of sleep disorders, depression and dementia which are here related with a more extended involvement of monoamine nuclei of the lateral RF as well as of the median raphe nuclei. **(C)** Severe *SNCA* point mutations and large gene rearrangements—They present as a predominance of non-motor autonomic, psychotic and cognitive symptoms relying on the massive involvement of rostral and caudal areas of the RF extending above and beyond the brainstem.

In the process of re-defining Parkinson’s disease (PD) a task force is working on various symptoms which, despite being unrelated to the extra-pyramidal motor system, are now considered as fundamental features of PD (Poewe, 2008; Fornai and Ruggieri, 2013; Marras and Chaudhuri, 2016; Wei et al., 2016). Although most cardinal symptoms of PD involve the extra-pyramidal motor system, an in depth knowledge of PD patients led to describe a variety of non-motor alterations as well as pyramidal motor dysfunctions which are presently under intense scrutiny (Li et al., 2010; Fornai and Ruggieri, 2013; Fornai et al., 2013; Natale et al., 2013; Xu et al., 2015; Zou et al., 2016). In fact, the occurrence of these symptoms may be helpful to discern between various PD genotypes/phenotypes, while it provides new vistas on the variety of brainstem reticular nuclei, which may or may not, be recruited during the disease course (Chao et al., 2015). Despite being missed out for a long time or being considered as an unexpected complication of a pure extra-pyramidal motor disorder, non-motor symptoms (NMS) are now a fundamental feature of PD. At the same time NMS provides an unusual perspective to elucidate the anatomical network sub-serving a brainstem monoamine disorder (BMD) which is the anatomical core of PD. In PD, NMS are often present during the disease course (Chaudhuri et al., 2006; Li et al., 2010; Bonnet et al., 2012; Chege and McColl, 2014; Bastide et al., 2015; Gao et al., 2015). In fact, brain areas, which control extra-pyramidal motor systems concomitantly, affect other neurological and psychiatric domains. This is evident when examining several reticular brainstem nuclei, whose functions affect both extra-pyramidal motor circuitries and a variety of non-motor domains during the neuropathology of sporadic PD (S-PD; Fornai and Ruggieri, 2013). Within these sub-cortical regions a variety of activities beyond extra-pyramidal motor control take place, which explains why non-motor alterations should be expected to occur rather than being unusual in most PD patients. In a recent article, we emphasized such a concept focusing on the involvement of a number of brainstem monoamine nuclei (Fornai and Ruggieri, 2013). In this analysis, we proposed the definition of BMD as more balanced to define the neuroanatomy of PD. In fact, a constellation of nuclei belonging to the brainstem reticular formation (BRF), are affected at various disease/stage severity. There is an appreciable site-specificity, which connects neuroanatomy with the onset of NMS and this works as a general model to build a clinical anatomical correlation within various PD syndromes. Within this context, progress in neurogenetics provided a powerful tool to improve PD nosography since specific gene/protein alterations connect quite specifically with clusters of both motor and NMS, which in turn, are related to a damage in quite selective brain areas. This configures PD as a spectrum of brainstem disorders which ranges between the occurrence of solely extra-pyramidal motor symptoms, up to the coexistence of pyramidal, extra-pyramidal symptoms along with NMS. Remarkably, a deep insight in the genetics of PD, along with the progressive awareness of NMS in PD, provided two key elements, which fostered the need to re-define PD itself. This is listed in the form of three paradigms shown in Figure 1 as PD due to mutations in three different loci (*Parkin; LRRK2; SNCA*). Similarly, Figure 2 shows the anatomy of the BRF and highlights those reticular nuclei which are often recruited in each paradigm of PD (Parkin; LRRK2; SNCA). When combining Figures 1, 2, one may get a general correlation between PD genotype, the occurrence of specific NMS, and the recruitment of specific brainstem reticular nuclei. For instance, Parkin disease represents an almost pure extra-pyramidal motor disorder. This is well described by Doherty et al. (2013) showing the paucity of symptoms (Figure 1) and affected brain areas (Figure 2), limited to the substantia nigra pars compacta (SNpc) dorsal tier, locus coeruleus (LC) and dorsal motor nucleus of the vagus. Conversely, the specific point mutation p.G51D in the *SNCA* gene (Lesage et al., 2013) or multiplications of *SNCA* gene itself leads to a plethora of non-motor alterations (Figure 1). In this latter SNCA-dependent syndrome, motor alterations surpass the extra-pyramidal circuitry featuring also a pyramidal disorder with fatal prognosis (Mutez et al., 2011; Chen et al., 2015; Kiely et al., 2015). The p.G51D mutation additionally shifts the disease towards multiple system atrophy (MSA). The great amount of NMS shown in Figure 1 is consistent with massive recruitment of brainstem nuclei shown in Figure 2. Roughly, in the middle of these extremes we describe most *LRRK2*-associated PD, which features extra-pyramidal motor symptoms along with some NMS (Zimprich et al., 2004). This resembles most *SNCA* point mutations but p.G51D, as well as S-PD patients (Figure 1).

## The Case of Parkin Disease

The *Parkin* gene (OMIM 602544), codes for a protein called Parkin, a E3 ubiquitin-ligase, which tags altered proteins by ubiquitin chains (Hristova et al., 2009; Yoshii et al., 2011). The loss of function of parkin activity leads to a restricted pathology of the mesencephalic RF (SNpc; Takahashi et al., 1994), which is affected only in the ventral tier (Doherty et al., 2013). This is quite odd in PD since we are now aware that in most PD cases the pontine nucleus of LC (A6) is massively affected and represents a hallmark for pathological diagnosis (Braak et al., 2000; Dickson, 2012). Mutations in *Parkin* produce autosomic recessive (AR) parkinsonism with early onset. Alterations are spread over the entire gene and include deletions and duplications of one or more exons in more than 50% of cases (Matsumine et al., 1997; Kitada et al., 1998; Abbas et al., 1999; Labandeira-Garcia et al., 2011). Motor symptoms of PD patients with two *Parkin* mutations present with classic parkinsonism, slow disease progression and more symmetrical onset, with fewer NMS than classic PD (Lesage and Brice, 2009; Lohmann et al., 2009; Kasten et al., 2010), supporting the concept of limited pathology (Kägi et al., 2010). In line with this, the frequency of dementia in *Parkin* mutations matches the frequency of the general population above the age of 65 (Khan et al., 2003; Macedo et al., 2009; Xu et al., 2012). Even psychiatric symptoms are almost absent (Lohmann et al., 2009; Alcalay et al., 2014). These observations may be unexpected since the occurrence of psychiatric disorders PD patients is connected with dopamine (DA)-induced motor fluctuations. Indeed, these patients undergo DA-dependent motor fluctuations early in their life, nonetheless these motor fluctuations do not contaminate the psychiatric domain. This evidence is critical to understand the neurobiology of DA-dependent psychiatric disorders. The occurrence of fluctuations in response to DA replacement therapy is concomitant with a massive loss of DA axons (Lohmann et al., 2009). Thus, the DA replacement therapy generates peaks and valleys of extracellular DA concentrations, which trigger a non-canonical transduction pathway within post-synaptic neurons (Gerfen, 2006; Biagioni et al., 2009). When the DA axons are present, the DA substitution therapy does not generate fluctuations. In fact, the excess of DA concentrations is quickly buffered by the powerful uptake from surrounding DA terminals via the DA transporter (DAT). In PARK2 patients, the loss of DA selectively occurs in the dorsal striatum which produces motor fluctuations only consistent with the loss of ventral tier of the SNpc. In contrast, the meso-limbic DA rising from the reticular nucleus of ventral tegmental area (VTA) pathways is spared preserving DA fluctuations in the ventral striatum and brain areas, which are key for psychopathology. Other psychiatric symptoms such as depression, panic, and anxiety are absent as well, which is likely to depend on sparing 5-hydroxytryptamine (5-HT) and the NA neurons of the RF (Figure 2; Fornai et al., 2013). In fact, depressive symptoms in PD as a whole syndrome, or a few items such as apathy or anhedonia, are likely to rely on degeneration of reticular 5-HT dorsal raphe nucleus and/or NA LC nucleus (Politis and Loane, 2011). Interestingly, although *PINK1* mutation carriers are clinically indistinguishable from *Parkin* mutation carriers, frequency of depression is higher in *PINK1* carriers, calling for more detailed anatomical comparisons between these two diseases (Schneider and Klein, 2010; Ricciardi et al., 2014; Chaudhuri et al., 2015). Autonomic dysfunction often relies on medullary A1, A2 and C1 and C2 noradrenergic and adrenergic neurons, respectively, which provide the descending fibers to preganglionic sympathetic neurons (Figure 2). The absence of disease spreading caudally may explain the preservation of blood pressure, micturition, sweating and other autonomic functions in PARK2 patients (Palma and Kaufmann, 2014). This is further explained by the lack of involvement of post-ganglionic ortho-sympathetic neurons, which otherwise degenerate in most PD cases. The lack of upstream disease progression and preservation of cholinergic and noradrenergic ascending reticular pathways as shown in Figure 2 may explain why cognitive dysfunction and sleep disorders are absent in these patients. As reported for *PINK1* and *DJ-1*, these PD patients show a largely preserved sense of smell, adding evidence to the observations that autosomal dominant forms of monogenic parkinsonism exhibit more severe olfactory impairment than the recessive ones (Yoritaka et al., 2011). Only in a few cases slight psychiatric symptoms may occur in Parkin PD patients (Cooney and Stacy, 2016; Lynch and Fujioka, 2016).

## LRRK2 Mutations

Mutations of the *LRRK2* gene (leucine-rich repeat kinase 2, OMIM 609007) is considered the most common genetic cause of late-onset, autosomal-dominant (AD) familial PD (PARK8; Paisán-Ruíz et al., 2004). The gene codes for a member of the leucine-rich repeat kinase family, widely expressed in isocortex, striatum, cerebellum, BRF and hippocampus (Sweet et al., 2015).

Several *LRRK2* mutations have been reported so far. These genotypes differently affect the amount of catecholamine nuclei of the BRF, and produce various NMS patterns (Vitte et al., 2010). *LRRK2* overlaps with alpha-synuclein pathology in the BRF, where deposition of LRRK2 appears to anticipate alpha-synuclein pathology (Alegre-Abarrategui et al., 2008). This makes the involvement of brainstem reticular nuclei in LRRK2 PD closely resembling point *SNCA* mutations and idiopathic PD.

In fact, clinical data support that in LRKK2 patients the prevalence of NMS is similar to idiopatic PD (i-PD; Estanga et al., 2014; Gaig et al., 2014).

Depression is quite common and can affect up to 40% of individuals with *LRRK2* mutations (Chaudhuri et al., 2006; Langston, 2006; Schrag and Schott, 2006; Shanker et al., 2011; Gaig et al., 2014; Pont-Sunyer et al., 2015). Similarly, REM sleep behavioral disorder (RBD) is quite frequent (Trinh et al., 2014). This suggests a recruitment of ascending reticular nuclei extending beyond the SNpc. In fact, just like idiopathic PD, LRRK2 involves the substantia nigra and LC (Vitte et al., 2010).

The occurrence of dementia in 17% of LRKK2 patients is lower than i-PD (Aarsland and Kurz, 2010). This appears to rely on altered synaptic activity within hippocampus. Supporting this concept, transgenic mice expressing p.G2019S *LRRK2* mutations show altered long-term depression (LTD) with potential impairment of learning and memory as shown in PD patients carrying *LRRK2* mutations (Shanker et al., 2011; Sweet et al., 2015). Olfactory functions is preserved in PD patients carrying the *LRRK2* mutations which is confirmed in preclinical models where mice expressing p.R1441C *LRKK2* mutations exhibit normal olfactory function (Tsika et al., 2014). These latter findings may be in contradiction with the classic Braak staging of PD. Nonetheless, the brainstem progression involves monoamine containing nuclei of the RF and starts from the dorsal nucleus of the vagus and nucleus solitarius (Kingsbury et al., 2010). According to Figure 2 this area corresponds roughly to the A2/C2 catecholamine region of the medulla. The cortical pathology is independent from the recruitment of the brainstem and so would be the olfactory allocortex including the olfactory bulb.

## Slight and Severe SNCA Point Mutations and Multiplications

The *SNCA* gene (alpha-synuclein, OMIM 163890) was the first gene to be associated with familial parkinsonism (Houlden and Singleton, 2012). This gene codes for the protein alpha-synuclein which is altered in both familial PD (F-PD) and S-PD (Oczkowska et al., 2013). Both point mutations (PARK1) and gene multiplications (PARK4), have been reported to produce PD with different age at onset, penetrance and clinical motor and non-motor features (Singleton et al., 2003; Puschmann et al., 2009; Kiely et al., 2013).

### SNCA Multiplications

Triplication carriers have disease onset about 10 years earlier and a more rapid disease course than duplication carriers, who overall closely resemble i-PD patients, while higher clinical variability has been reported for different point mutations (Miller et al., 2004; Ferese et al., 2015). NMS are constantly present in all SNCA patients. Remarkably, those patients carrying four copies of *SNCA* undergo a severe phenotype of PD that leads to death in a few years from diagnosis. These patients possess early onset sleep disorders, autonomic dysfunction, and psychotic episodes, with massive involvement of the BRF (Figure 2). This is evidenced by a massive deposition of alpha- synuclein oligomers in the BRF (Roberts et al., 2015). These oligomers are made up of prion-like partially proteinase-K resistant alpha-synuclein just like oligomers in the brainstem of PD patients. These patients show rapid cognitive and motor deterioration which surpasses the extrapyramidal circuitry affecting the corticospinal pathway as evidenced by a pyramidal syndrome which adds on the extrapyramidal movement disorder which is the main cause of death in these patients (Singleton et al., 2003; Ferese et al., 2015). These latter syndromes indicate that neuronal degeneration recruits brain areas above the brainstem level extending well beyond the classic neuropathology described by Tretiakoff in 1919 (Parent and Parent, 2010).

### SNCA Duplication

*SNCA* duplications lead to various NMS (dysautonomia, RBD, hallucinations, depression; Konno et al., 2016) albeit with lower prevalence compared with carriers of four *SNCA* copies, but still higher compared with i-PD. *SNCA* duplication shows an incomplete penetrance in some carriers and, interestingly, NMS are reported in both healthy people and PD patients carrying this kind of mutation. Such carriers are reported to show an impairment of reward learning, suggesting that a copy number variation of the *SNCA* gene may be associated with NMS (especially selective learning impairment) more than producing classic motor parkinsonism (Kéri et al., 2010). In contrast, olfactory dysfunction and RBD are observed only in symptomatic carriers (Nishioka et al., 2009).

### SNCA Point Mutations

High clinical variability has been reported in *SNCA* point mutations, where NMS are less frequent if compared with *SNCA* multiplications, and dementia is also less frequent when compared with i-PD patients (Aarsland et al., 2005; Aarsland and Kurz, 2010). NMS features are variably associated with different point mutations. p.A53T and p.A30P are reported to correlate with dementia (Puschmann et al., 2009), p.E46K with dementia and visual hallucinations (Zarranz et al., 2004), and p.G209A with olfactory dysfunction and RBD. In G209A carriers, prominent motor decline and deterioration of autonomic and cognitive function occur.

Remarkably, the point mutation p.G51D leads to a rapidly evolving syndrome. In this case, despite no gene multiplication occurs, there is a widespread neuropathology that, similarly to patients carrying four *SNCA* copies, leads to the involvement of the pyramidal tract. Even in this case, the cortico-spinal degeneration is the main cause of death with a clinical course and neuropathology which surpasses PD and features MSA, where a plethora of NMS and massive areas in the brainstem are affected to extend rostrally to the prosencephalon and caudally to the spinal cord (Lesage et al., 2013; Kiely et al., 2015).

## Conclusions

The occurrence of both motor NMS in most cases of PD is now well established. This is related to the engagement of a constellation of brain areas mostly located in the core of the BRF. The severity and variety of motor symptoms and mostly the various occurrence of NMS produce different PD syndromes, which appear more and more as a variety of diseases. This is substantiated by the number of genetic alterations, which may produce PD, and it is confirmed by the variety brain nuclei which may be involved. The present manuscript suggests a clinical anatomical correlation based on different genetic alterations which produce PD. These genetic conditions were used as paradigms for a clear-cut separation of different PD syndromes. The various involvement of brainstem reticular nuclei in these three paradigms was discussed.

Evidence was provided that the onset of an almost pure extrapyramidal syndrome due to a *Parkin* mutation was related to a quite unusual pathology mostly confined to the ventral tier of the SNpc. On the other hand, the multiplications of the *SNCA* genes and the p.G51D *SNCA* point mutation were related to the most severe phenotype in which a plethora of NMS (psychotic and mood disorder, sleep disorder, cognitive alterations, autonomic dysfunctions) were associated with a massive involvement of several nuclei of the BRF. Remarkably the most severe condition (point mutation p.G51D is definitely considered as MSA rather than PD). In the middle of these paradigms, we described the clinical anatomical correlation in the case of most *LRRK2* mutation and *SNCA* point mutation, which resembles S-PD. An interesting correlation was evident between specific NMS and specific brainstem reticular nuclei. This is quite remarkable for anxiety, mood and sleep disorders which associate with serotonergic neurons and lateral reticular noradrenergic nuclei. Similarly, the occurrence of autonomic dysfunctions witnesses for the involvement of the bulbar noradrenergic and adrenergic areas (A1/A2 and C1/C2, respectively). The impairment of oculo-motor activities suggests the impairment in the paramedianpontine RF, while the presence of DA-dependent psychotic symptoms relates with the involvement of the mesencephalic VTA of the RF which projects to limbic and iso-cortical regions. In the most severe phenotypes, the disease surpasses the definition of BMD and configures as a MSA where the early involvement of the brainstem is expected to spread quickly to distant brain regions also including the motor cortex and spinal cord. The spreading of symptoms in the disease course is likely to rely on the prion-like properties of key proteins such as alpha-synuclein. The variety of brain areas is not totally unexpected in the light of the remarkable branching and collateralization of the iso-dendritic neurons forming the core of the BRF which represents a powerful stream to drive physiological and altered synaptic activity.

## Author Contributions

SG: coordination and writing the review. RF: pubmed research and state of the art about genetic of parkin. FB: pubmed research and state of the art about genetic of *LRRK2*. CLB: pubmed research and state of the art about genetic of *SNCA*. RC: participated in critically revising the article. AMPG: participated in drafting the article. FL: pubmed research and state of the art about genetic of *SNCA* duplication and triplication. GN: participated in critically revising the article for important intellectual content. MS: participated in drafting the article. FF: coordinating and writing the article. Participated in drafting the article. Participated in critically revising the article for important intellectual content.

## Conflict of Interest Statement

The authors declare that the research was conducted in the absence of any commercial or financial relationships that could be construed as a potential conflict of interest.
